# Phenotypic maps for precision medicine: a promising systems biology tool for assessing therapy response and resistance at a personalized level

**DOI:** 10.3389/fnetp.2023.1256104

**Published:** 2023-10-25

**Authors:** Sayantan Bhattacharyya, Shafqat F. Ehsan, Loukia G. Karacosta

**Affiliations:** ^1^ Department of Cancer Systems Imaging, University of Texas MD Anderson Cancer Center, Houston, TX, United States; ^2^ Department of Radiation Oncology, University of Texas MD Anderson Cancer Center, Houston, TX, United States

**Keywords:** personalized medicine, phenotypic maps, therapy resistance, tumor heterogeneity, multi-omics, systems biology, computational biology

## Abstract

In this perspective we discuss how tumor heterogeneity and therapy resistance necessitate a focus on more personalized approaches, prompting a shift toward precision medicine. At the heart of the shift towards personalized medicine, omics-driven systems biology becomes a driving force as it leverages high-throughput technologies and novel bioinformatics tools. These enable the creation of systems-based maps, providing a comprehensive view of individual tumor’s functional plasticity. We highlight the innovative PHENOSTAMP program, which leverages high-dimensional data to construct a visually intuitive and user-friendly map. This map was created to encapsulate complex transitional states in cancer cells, such as Epithelial-Mesenchymal Transition (EMT) and Mesenchymal-Epithelial Transition (MET), offering a visually intuitive way to understand disease progression and therapeutic responses at single-cell resolution in relation to EMT-related single-cell phenotypes. Most importantly, PHENOSTAMP functions as a reference map, which allows researchers and clinicians to assess one clinical specimen at a time in relation to their phenotypic heterogeneity, setting the foundation on constructing phenotypic maps for personalized medicine. This perspective argues that such dynamic predictive maps could also catalyze the development of personalized cancer treatment. They hold the potential to transform our understanding of cancer biology, providing a foundation for a future where therapy is tailored to each patient’s unique molecular and cellular tumor profile. As our knowledge of cancer expands, these maps can be continually refined, ensuring they remain a valuable tool in precision oncology.

## Introduction

Therapy resistance remains the major culprit of cancer related mortality (Vasan et al., 2019). Overcoming resistance poses a significant challenge given the intra- and inter-heterogeneity of tumors and the plastic nature of therapy resistant phenotypes that develop over time during treatment (Zhu et al., 2021). The scenario gets more complicated when tumor microenvironment (TME) elements are considered (Mintz and Illmensee, 1975; Park et al., 2000). Interactions between tumor cells with the surrounding extracellular matrix and associated cells, resident microbiota and immune cell populations drive phenotypic alterations that often augment metastatic and therapy resistance potential of malignant cells (Neophytou et al., 2021). The extraordinary heterogeneity tumors exhibit both within the same patient spatially, but also between patients, highlights the necessity of implementing personalized medicine (Proietto et al., 2023). Achieving precision oncology and personalized medicine necessitates the implementation of cancer systems approaches that often combine single-cell multi-omic technologies and advanced computational tools.

Although the concept of personalized medicine has already been implemented in some types of cancers including breast (e.g., ER, PR and Her2 expression status), lung (e.g., EGFR) and colorectal (e.g., KRAS) (Hoeben et al., 2021), screening/targeting for only a handful of markers remains insufficient for personalized medicine throughout the course of treatment, given that it fails to address the overall heterogeneous and dynamic nature of tumors (Asleh et al., 2022). Since therapy response for an individual varies greatly based on an array of genomic, epigenomic and proteomic pathways, marker-based classification for precision medicine requires in-depth knowledge and understanding of the functional plasticity of cancer cells and the TME as a whole. Therapeutic failure correlates with a variety of events; pre-existing mutations that are “favorably selected” by therapy, therapy-induced phenotypic changes (Sharma et al., 2010; Shaffer et al., 2017; Salgia and Kulkarni, 2018), and alterations of the TME, triggered by genomic mutations (Zhang et al., 2022). Traditionally, validation of novel targetable markers has been achieved by using knock out murine models, however these models fail to address marker bias, heterogeneity, and dynamic changes at the single-cell level (Wellner et al., 2009; Zheng et al., 2015). The dynamic nature of therapy resistance is often overlooked, hence, it is paramount to increase our efforts in analyzing therapy resistant phenotypes directly in clinical specimens at the single-cell level longitudinally (Qin et al., 2020). Therefore, multi-omic approaches and leveraging better suited patient-derived models that take into account time-related changes within the TME are critical for achieving precision therapy (Xu et al., 2021).

The more we study tumor heterogeneity and phenotypic plasticity with state-of-the-art technologies, the more it becomes evident that they hold the clues to strengthen precision medicine efforts. Ability to understand the disease’s single-cell nature, at various time points per patient, will assist efforts in assessing and predicting therapy response at personalized level (Mundi et al., 2023). It is our belief that despite all the technological and computational advances researchers have in their arsenal today, the field is still lacking when it comes to developing applicable tools for personalized medicine. Specifically, we believe that constructing well-informed reference maps of therapy resistant states observed both in *in vitro* systems and in clinical specimens will become one of the main avenues through which we can achieve therapy response assessment and prediction one patient at a time.

In this perspective, we discuss how instrumental systems biology approaches and omics are to achieving precision oncology with a focus on constructing reference phenotypic maps for assessing and predicting therapy response at a personalized level. We highlight the PHENOtypic STAte MaP (PHENOSTAMP) (Karacosta et al., 2019) as a proof of concept example of how single-cell omics and machine learning tools can be utilized to build translational tools for assessing metastatic and therapy resistant phenotypes in clinical specimens and discuss future directions towards achieving individualized medicine.

### The era of omics and systems biology: tools and paths towards achieving personalized medicine

The shift towards the era of omics and systems biology signifies a new dawn in cancer research. The groundbreaking fusion of genomics, transcriptomics, proteomics, epigenomics, and metabolomics, and the holistic methodology of systems biology ushers in an innovative approach to understanding oncogenesis and therapy resistance (Williams et al., 2022). This transformative shift is grounded in advanced computational methodologies and bioinformatics toolkits, enabling the examination of cancer in a broader, more holistic context that transcends the constraints of the traditional reductionist approach (Heo et al., 2021), where complex biological systems are typically broken down into their components and analyzed individually (Friboulet and Thomas, 2005). Although reductionist approaches have led to great advances in biomedical sciences, studying individual components of a system are insufficient for understanding how a system operates in its entirety. By making sense of complex systems in their entirety, we are now at the forefront of “personalized medicine using panomics” - an emerging field that tailors treatment based on individial variability beyond just genetic variabilities (Langreth and Waldholz, 1999; Pfohl et al., 2021; Veenstra, 2021).

Single-cell sequencing, a remarkable breakthrough in the realm of genomics and transcriptomics, allows for comprehensive examination of the genome and transcriptome at an unparalleled level of detail (Li and Wang, 2021). Spatial genomic and transcriptomic technologies, exemplified by slide-DNA-seq (Zhao et al., 2022) and studies that interrogate spatial clonal copy number variations (CNVs) in the microenvironment of benign and malignant tissues (Erickson et al., 2022), as well as VISIUM, CosMX and GeoMX, further enhance this detailed examination by incorporating spatial information, effectively bridging the gap between bulk and single-cell sequencing (Ståhl et al., 2016; Merritt et al., 2020; He et al., 2022; Williams et al., 2022). Advancements in single-cell proteomics, specifically Cytometry by Time-Of-Flight (CyTOF), have further augmented our ability to dissect the TME at a cellular and phenotypic level (Bendall et al., 2012). These high-resolution techniques, when coupled with cutting-edge bioinformatics clustering and trajectory algorithms, provide unprecedented insights into the complex and dynamic nature of intratumoral heterogeneity (Friboulet and Thomas, 2005). Simultaneously, the evolution of multiplex imaging technologies, including among others imaging mass cytometry (IMC), multiplexed ion beam imaging (MIBI), cyclic immunofluorescence (cycIF), and Opal, have amplified our ability to dissect the TME at the spatial proteomic level (Tóth and Mezey, 2007; Lin et al., 2016; Gorris et al., 2018; Keren et al., 2018; Ptacek et al., 2020; Karacosta, 2021). These approaches facilitate the simultaneous detection of multiple biomarkers within a single tissue section, preserving the spatial context of cells and their interactions within the TME (Gorris et al., 2018). Finally, as we increasingly appreciate the role of the epigenome on phenotypic plasticity and drug resistance, methods for DNA methylation analysis (e.g., MeDIP-seq and WGBS) and histone modifications or nuclear organization (e.g., ChIP-seq, ChIA-PET, scATAC-seq) become critical tools in multi-omic systems biology approaches (Staunstrup et al., 2016; Flebbe et al., 2019; Zhang et al., 2019; Zhao et al., 2020; Mehrmohamadi et al., 2021; Kim et al., 2022). The high-dimensional data generated by these techniques, when subjected to machine learning algorithms, can unravel the complex spatio-temporal interactions and patterns within the TME that may be predictive of tumor progression and therapy response (Stack et al., 2014; Ahmed et al., 2022).

Computational biology has ushered in an array of novel bioinformatics tools that work in synergy with systems-based methodologies (e.g., network-based analyses, pathway simulations and machine learning models, tailored to untangle gene regulatory networks and signaling cascades (Sharma et al., 2010)). EcoTyper, a cutting-edge computational tool, is one such innovative development that facilitates high-throughput ecological analyses of the TME (Luca et al., 2021; Steen et al., 2021). Large-scale multi-omics databases such as The Cancer Genome Atlas (TCGA), Kyoto Encyclopedia of Genes and Genomes (KEGG) and the Genomic Data Commons (GDC) have emerged as indispensable resources (Kanehisa and Goto, 2000; Weinstein et al., 2013). These repositories, packed with rich genomic, transcriptomic, and proteomic data, provide robust platforms for integrative and systems-level analyses. These databases serve as the bedrock for developing robust prognostic and predictive models, thereby accelerating the shift towards a more personalized, data-driven approach to cancer treatment. Table 1 highlights a selection of multi-omics databases and bioinformatics pipelines suitable for generating high-resolution reference maps, from the plethora available.

**TABLE 1:** Highlights a selection of state-of-the-art techniques, multi-omics databases and bioinformatics pipelines that can be used for constructing reference maps for personalized medicine. See text for additional details.

	MuIti-omics databases	Bioinformatics pipelines	Relevance to phenotypic mapping	References
Spatial Transcriptomics	TCGA, GEO	Seurat, spatiaLDE	High-throughput spatial resolution	Weinstein et al. (2013), Satija et al. (2015), Clough and Barrett (2016), Svensson et al. (2018)
scRNA-seq	SRA, ENA	STAR, Cell Ranger, Bowtie2, Kallisto	Single-cell gene expression profiling	Leinonen et al. (2011), Langmead and Salzberg (2012), Dobin et al. (2013), Bray et al. (2016), Zheng et al. (2017)
CNV Analysis	COSMIC, dbVar, DGVa	GATK, CNVnator	Detecting large scale genomic alterations	Abyzov et al. (2011), Van der Auwera et al. (2013), MacDonald et al. (2014), Tate et al. (2019)
scATAC-seq	GEO, SRA	Cicero, ArchR	Single-cell chromatin accessibility	Clough and Barrett (2016), Leinonen et al. (2011), Tian et al. (2020), Granja et al. (2021)
ChIP-seq	ENCODE, SRA	MACS, Bowtie2	Identification of transcription factor binding sites	Leinonen et al. (2011), Langmead and Salzberg (2012), ENCODE Project Consortium (2012), Zhang et al. (2008)
Metabolomics	MetaboLights, GNPS	XCMS, MetaboAnalyst	Small molecule profiling	Smith et al. (2006), Xia et al. (2009), Haug et al. (2013), Wang et al. (2016)

### The concept of systems-based mapping of tumor heterogeneity

The idea of “mapping” is not new. Since the beginning of medical sciences, researchers have tried to generate the “grammar” of human’s microscopic anatomy and cellular makeup as well as their changes in various disease backgrounds. However, a map’s accuracy relies on the amount of detailed information each map is based on. With the recent advancements in systems-based biology and big data repositories, personalized medicine can now become a reality and the concept of mapping plays a pivotal role in it. In recent years, comprehensive maps or “atlases” of single-cell resolution of normal tissue/organs and diseases (including cancers) have been assembled by using multi-omic data integration (Azizi et al., 2018; Friebel et al., 2020; Kuett et al., 2021; Hansen et al., 2022). Some of these studies and proposed approaches focus on tumor spatial and phenotypic heterogeneity specifically (Liu et al., 2010; Heindl et al., 2015; Patkulkar et al., 2023). These maps do an excellent job of portraying in great detail the underlying molecular, cellular make up and heterogeneity of the tissue and help better classify diseases and cancer types. However, they have not been developed to be used as translational tools *per se* where one could utilize them for directly analyzing and assessing newly acquired clinical specimens, both visually and functionally as in the PHENOSTAMP example that will be discussed in the following section.

A key component of constructing detailed reference maps, is the acquisition and analysis of clinical specimens, which is also a main limiting factor given the difficulties in obtaining them in large enough numbers and amount for data acquisition and downstream analyses. However, recent advances in single-cell technologies have surpassed this obstacle with their multimodal approaches. Today a small histo-section from a tumor can generate spatial genomics map for clones of ductal carcinoma of the breast (Lomakin et al., 2022), whereas three-dimensional multiplex imaging can shed light on the tissue microenvironment using the principle of mass cytometry (Kuett et al., 2021).

The variety of clinical specimens should extend beyond just the abundance of solid tumors. Other sources such as circulating tumor cells (CTCs), malignant cells isolated from pleural effusions, and pre-cancerous cells obtained from cytobrush swabs, also hold significant potential. They can generate extensive, high-dimensional data for each patient, whether it is through single-cell profiling or by creating and studying intricate patient-derived tumor organoids at later time-points (Tsao et al., 2018; De Luca et al., 2021; Sorolla et al., 2021; Gaw and Gribben, 2022; Hsu et al., 2023). Furthermore, integrating two or more different modes of high throughput analyses of the same model has been proven to provide the necessary information for improved patient outcome prediction (Zhang et al., 2021; Ebisudani et al., 2023).

Acquiring high-dimensional data carries the potential to discover specific patterns in each tumor that can be used for creating personalized maps. These maps can be used to predict treatment outcome and decipher the individual tumor’s functional heterogeneity at the route to therapy induced phenotypic plasticity and resistance. The single-cell-based approach on collecting big data from a heterogenous mass of quandary and the subsequent generation of metrices from them is undoubtedly challenging yet promises to be rewarding at the same time (Kashyap et al., 2022). Depending on the type of data and models used for map constructing, one can envision different types of mapping that convey information on specific biological phenomena that drive tumor progression and therapy resistance. Imaging-based marker analysis in 3D from a chemoresistant patient-derived tumor organoid will have the ability to map the spatial correlation between tumor with its microenvironment and their cumulative contribution on resistance (Lukonin et al., 2021; Van Hemelryk et al., 2023). Integrating single-cell RNAseq data with genome mapping and CyTOF analyses from longitudinal clinical specimens has the power to holistically map out the development of a patient’s therapy resistance from multiple aspects (mutational, transcriptional, proteomic level). Time-lapse-based single-cell analysis across different cancer stages will be able to generate the branching routes of clonal evolution and the dynamics of phenotypic plasticity (Coffey et al., 2013; Randriamanantsoa et al., 2022; Desjardins-Lecavalier et al., 2023). The idea of mapping can be diverse based on an observer’s perspective, and it can be molded in various ways once the cancer’s structural and functional heterogeneity has been explored in a detailed manner. Ultimately, the more data we can acquire/generate from *in vitro*/*ex vivo* models and clinical specimens, the better we can construct clinically applicable maps.

### Phenotypic maps: a computational vision for the future of precision oncology and the PHENOSTAMP example

In the evolving landscape of precision oncology, predictive maps have begun to emerge as a potential game-changing tool, signifying a transformative era in cancer research. It is paramount to construct maps that not only encompass high-dimensional, underlying biological complexity, but are also intuitively easy to use and understand for cancer researchers and clinicians alike. Only then can a map be truly translational and clinically applicable. For a phenotypic map to function as a translational tool, generation and acquisition of high-quality data from carefully designed experiments and clinical specimens that best reflect tumor heterogeneity and dynamic states that are visited during drug perturbations (i.e., observed phenotypic states) is critical. Subsequently, incorporating and “compacting” the high-dimensional data on, for example, a low dimensional 2D plane, creates a landscape of “observed states”, where each x y coordinate represents a phenotypic state that may reflect a metastatic or drug-specific resistant trait. Therefore, when using this map as a reference to analyze a new sample, the positioning of each cell of a tumor on the 2D plane can be used to assess the metastatic or drug-resistant properties of the overall cell population under study.

The PHENOtypic STAte MaP (PHENOSTAMP) program, exemplifies such innovative mapping approaches (Karacosta et al., 2019). It integrates high-dimensional data derived from advanced techniques like CyTOF to construct a comprehensive, visually intuitive map with single-cell proteomic resolution, that encapsulates the Epithelial-Mesenchymal Transition (EMT) and Mesenchymal-Epithelial Transition (MET) states in lung cancer cells. It is important to note, that there have been a number of studies that have focused on identifying and describing cancer EMT states, with not necessarily focusing on constructing a translational tool like the PHENOSTAMP program (Pastushenko et al., 2018; Brown et al., 2022; Burkhardt et al., 2022). For constructing PHENOSTAMP, authors used *in vitro* time course experimentation to induce EMT and MET in lung cancer cells, thus capturing intermediate transition states that when pieced together, represent the spectrum of EMT states, that are often associated with cancer aggressiveness and drug resistance (Kalluri and Weinberg, 2009; Schliekelman et al., 2015; Bakir et al., 2020). Additionally, the authors performed advanced computational analysis where they were able to estimate transition probabilities among the identified EMT and MET states. Therefore, by projecting a new specimen on PHENOSTAMP one can not only assess the heterogeneity of a given specimen at the given timepoint but can also theoretically predict what new states will be populated in the future during cancer progression and/or treatment. Importantly, PHENOSTAMP supports the projection of clinical samples onto this elaborate, reference EMT-MET map, enabling the characterization of their phenotypic profile with single-cell resolution in terms of *in vitro* EMT-MET analysis. This approach provides a nuanced characterization of clinical samples and may pave the way for evaluating the clinical relevance of EMT in future cancer studies. Such methods set the stage for a new era of personalized medicine, where treatment decisions could be based on the unique molecular profile of each patient’s tumor not only at time zero, but also during the course of therapy, by, for example, analyzing longitudinal liquid biopsies (Siravegna et al., 2017; Blackhall et al., 2018).

**FIGURE 1:**
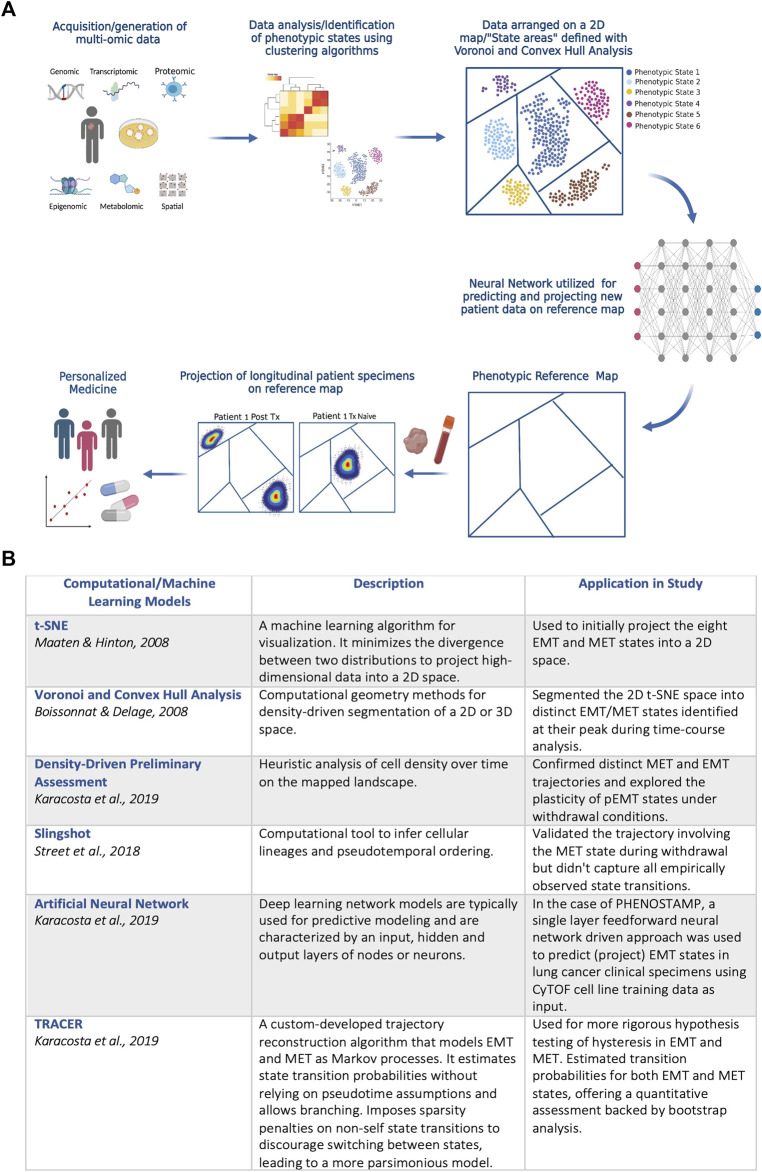
**(A)** Schematic diagram on the PHENOSTAMP (Karacosta et al., 2019) concept. The first step requires acquiring high-dimensional data that provide in depth views of the biological process under study. In the case of PHENOSTAMP, single-cell proteomic data (CyTOF) from lung cancer cell lines undergoing EMT and MET were generated from optimally designed time-series *in vitro* experiments. We propose that clinically applicable maps should incorporate multi-omic integrated data not only from *in vitro* experiments but also directly from clinical specimens and patient-derived organoids and models. Following data acquisition, dimensionality reduction and clustering analysis is utilized to define phenotypic states that malignant cells visit across cancer stages and/or perturbations (e.g., therapy). Unbiased analysis but also biological prior knowledge is required for optimal state definition. The next step is to decide best 2D representation of the acquired data for constructing a visually intuitive map. For PHENOSTAMP, t-SNE was used for 2D representation of the data, and Voronoi and Convex Hull analysis were utilized for breaking down the 2D map to the areas representing the various defined phenotypic states. Finally, a feedforward neural network is utilized for projecting new clinical specimens onto the reference map (in the case of PHENOSTAMP lung cancer clinical specimens were analyzed with CyTOF and the single-cell proteomic data were projected on the map and represented as density plots). Given that high-content data are used to construct a reference map, each state may indicate metastatic traits, drug resistant features and targetability and thus enables the user to assess/predict phenotypic heterogeneity, therapy response and resistance at a personalized level. Created with Biorender.com. **(B)** Descriptive summary of computational and machine learning models that were used in PHENOSTAMP. See text for more details.

PHENOSTAMP effectively highlights how utilizing systems biology approaches that include single-cell omic technologies and computational analysis can help navigate efforts towards personalized medicine, while also interrogating complex biological processes like EMT. Specifically, in the case of PHENOSTAMP, CyTOF was first used to obtain single-cell proteomic readouts of EMT-transitioning cells. Dimensionality reduction and clustering analysis were performed to identify EMT and MET states which were subsequently arranged on a 2D t-SNE (Maaten and Hinton, 2008) plane. The 2D plane was segmented using Convex Hull and Voronoi analysis (Boissonnat and Delage, 2005) to visually represent distinct EMT and MET areas (i.e., states) of the map. Finally, the authors used a feedforward neural-network driven approach, trained on the CyTOF cell line data used to construct PHENOSTAMP, to predict dynamic phenotypic EMT states of lung cancer cells in clinical specimens. Thus, PHENOSTAMP serves as a reference map, through which by projecting patient specimens onto it, one can visually and quantitatively assess EMT status of any given sample that is analyzed with CyTOF. The PHENOSTAMP example underscores how neural networks and machine learning approaches in general can be applied to multi-omic data, becoming more and more the main tools for predicting drug resistance and other clinical features (Liu et al., 2019). A schematic example of the PHENOSTAMP concept, and the computational tools and machine learning models that were utilized for its construction and function are shown in Figure 1.

## Discussion

As proof of concept, PHENOSTAMP is a great example on how one can build a translational tool for personalized medicine. However, as with any model, there are certain limitations. First, PHENOSTAMP was constructed using cell line data, that were then used to predict EMT states in clinical specimens. It is expected, that not all clinically applicable EMT states were “observed” and therefore not represented in PHENOSTAMP. Second, only proteomic data were used towards constructing PHENOSTAMP, which included bias in choosing markers that best characterize the EMT process. Incorporating multi-omic data towards building a map, promises to reflect holistically a tumor’s makeup that will undoubtedly be more translational in nature. Leveraging computational frameworks that harmonize multimodal omic data (Liu et al., 2020; Ma et al., 2022) will be pivotal towards building optimal phenotypic maps. Although PHENOSTAMP was developed for studying EMT, one can envisage building maps that interrogate other biological processes such as therapy resistance, metastatic ability, dormancy, or many of these combined. As multiplexed imaging platforms also rapidly advance, maps may also represent histoarchitectural patterns of cell types and phenotypes that hold predictive power towards cancer prognosis and therapy response.

For translational approaches like PHENOSTAMP, benchmarking is a necessity for establishing robustness and clinical applicability. Several layers of validation are imperative and may include: 1) Evaluation Metrics: metrics like high-precision and high-recall become especially critical in single-cell studies (Hicks et al., 2022); 2) Cross-Validation: robust k-fold or stratified cross-validation is essential due to the complexities inherent in single-cell data and multi-omic approaches (James et al., 2017; Vabalas et al., 2019); 3) Baseline Comparisons: comparing advanced algorithms like TRACER and density-driven segmentation against baseline or existing models provides a comprehensive understanding of their incremental advantages (Street et al., 2018; Karacosta et al., 2019); 4) Hyperparameter Tuning: fine-tuning the hyperparameters can significantly impact a map’s clinical utility (Bergstra and Bengio, 2012); 5) Real-world Validation: validation with patient-derived samples is crucial for confirming an algorithm’s translational value; 6) Computational Efficiency: given the time-sensitive nature of clinical decision-making, evaluating the computational efficiency of an algorithm used is paramount.

Ultimately, we believe that constructing clinically applicable reference maps for personalized medicine will require analyzing patient-derived samples and clinical specimens with a combination and integration of a variety of omic technologies and advanced computational tools. This underscores once again the notion of studying cancer at multiple levels of the *system* to better recapitulate what takes place in each patient individually. It is therefore paramount for the research community to focus not only on promoting multidisciplinary collaborations, but more importantly on also training the next-generation of scientists to be equally adept in both experimental, systems and computational biology.

Reference maps can be intrinsically dynamic and adaptable to new data. As our understanding of cancer biology expands and novel omics data emerge, these maps can be continually refined and updated, ensuring they remain cutting-edge tools in the fast-paced field of precision oncology. By consolidating various omic data, reference maps provide an integrated, multi-dimensional perspective of the tumor landscape. This comprehensive approach, bolstered by technologies like multiplexed imaging platforms and machine learning approaches, has the potential to revolutionize our understanding of tumor biology, specifically the mechanisms underpinning metastasis and therapy resistance. Most importantly, it can serve as a catalyst for the development of novel therapeutic interventions, thereby reshaping the future of individualized cancer treatment. It is up to researchers to now harness the power of systems biology to achieve precision medicine one patient at a time.

## References

[B1] Abyzov A. Urban A. E. Snyder M. Gerstein M. (2011). CN Vnator: an approach to discover, genotype, and characterize typical and atypical CNVs from family and population genome sequencing. Genome Res. 21, 974–984. 10.1101/gr.114876.110 21324876 PMC3106330

[B2] Ahmed R. Zaman T. Chowdhury F. Mraiche F. Tariq M. Ahmad I. S. (2022). Single-Cell RNA Sequencing with Spatial Transcriptomics of Cancer Tissues. Int. J. Mol. Sci. 23, 3042. 10.3390/ijms23063042 35328458 PMC8955933

[B3] Asleh K. Riaz N. Nielsen T. O. (2022). Heterogeneity of triple negative breast cancer: current advances in subtyping and treatment implications. J. Exp. Clin. Cancer Res. 41, 265. 10.1186/s13046-022-02476-1 36050786 PMC9434975

[B4] Azizi E. Carr A. J. Plitas G. Cornish A. E. Konopacki C. Prabhakaran S. (2018). Single-Cell Map of Diverse Immune Phenotypes in the Breast Tumor Microenvironment. Cell 174, 1293–1308. 10.1016/j.cell.2018.05.060 29961579 PMC6348010

[B5] Bakir B. Chiarella A. M. Pitarresi J. R. Rustgi A. K. (2020). EMT, MET, Plasticity, and Tumor Metastasis. Trends Cell Biol. 30, 764–776. 10.1016/j.tcb.2020.07.003 32800658 PMC7647095

[B6] Bendall S. C. Nolan G. P. Roederer M. Chattopadhyay P. K. (2012). A deep profiler’s guide to cytometry. Trends Immunol. 33, 323–332. 10.1016/j.it.2012.02.010 22476049 PMC3383392

[B7] Bergstra J. Bengio Y. (2012). Random Search for Hyper-Parameter Optimization. J. Mach. Learn. Res. 13, 281–305.

[B8] Blackhall F. Frese K. K. Simpson K. Kilgour E. Brady G. Dive C. (2018). Will liquid biopsies improve outcomes for patients with small-cell lung cancer? Lancet Oncol. 19, e470–e481. 10.1016/S1470-2045(18)30455-8 30191851

[B9] Boissonnat J.-D. Delage C. (2005). “Convex Hull and Voronoi Diagram of Additively Weighted Points,” in Algorithms – esa 2005 (Berlin, Heidelberg: Springer), 367–378. 10.1007/11561071_34

[B10] Bray N. L. Pimentel H. Melsted P. Pachter L. (2016). Near-optimal probabilistic RNA-seq quantification. Nat. Biotechnol. 34, 525–527. 10.1038/nbt.3519 27043002

[B11] Brown M. S. Muller K. E. Pattabiraman D. R. (2022). Quantifying the Epithelial-to-Mesenchymal Transition (EMT) from Bench to Bedside. Cancers 14, 1138. 10.3390/cancers14051138 35267444 PMC8909103

[B12] Burkhardt D. B. San Juan B. P. Lock J. G. Krishnaswamy S. Chaffer C. L. (2022). Mapping Phenotypic Plasticity upon the Cancer Cell State Landscape Using Manifold Learning. Cancer Discov. 12, 1847–1859. 10.1158/2159-8290.CD-21-0282 35736000 PMC9353259

[B13] Clough E. Barrett T. (2016). The Gene Expression Omnibus database. Methods Mol. Biol. Clifton N. J. 1418, 93–110. 10.1007/978-1-4939-3578-9_5 PMC4944384 27008011

[B14] Coffey S. E. Giedt R. J. Weissleder R. (2013). Automated analysis of clonal cancer cells by intravital imaging. IntraVital 2, e26138. 10.4161/intv.26138 PMC3859690 24349895

[B15] De Luca A. Gallo M. Esposito C. Morabito A. Normanno N. (2021). Promising Role of Circulating Tumor Cells in the Management of SCLC. Cancers 13, 2029. 10.3390/cancers13092029 33922300 PMC8122820

[B16] Desjardins-Lecavalier N. Annis M. G. Nowakowski A. Kiepas A. Binan L. Roy J. (2023). Migration speed of captured breast cancer subpopulations correlates with metastatic fitness. J. Cell Sci. 136, jcs260835. 10.1242/jcs.260835 37313743 PMC10657211

[B17] Dobin A. Davis C. A. Schlesinger F. Drenkow J. Zaleski C. Jha S. (2013). STAR: ultrafast universal RNA-seq aligner. Bioinformatics 29, 15–21. 10.1093/bioinformatics/bts635 23104886 PMC3530905

[B18] Ebisudani T. Hamamoto J. Togasaki K. Mitsuishi A. Sugihara K. Shinozaki T. (2023). Genotype-phenotype mapping of a patient-derived lung cancer organoid biobank identifies NKX2-1-defined Wnt dependency in lung adenocarcinoma. Cell Rep. 42, 112212. 10.1016/j.celrep.2023.112212 36870059

[B19] ENCODE Project Consortium (2012). An integrated encyclopedia of DNA elements in the human genome. Nature 489, 57–74. 10.1038/nature11247 22955616 PMC3439153

[B20] Erickson A. He M. Berglund E. Marklund M. Mirzazadeh R. Schultz N. (2022). Spatially resolved clonal copy number alterations in benign and malignant tissue. Nature 608, 360–367. 10.1038/s41586-022-05023-2 35948708 PMC9365699

[B21] Flebbe H. Hamdan F. H. Kari V. Kitz J. Gaedcke J. Ghadimi B. M. (2019). Epigenome Mapping Identifies Tumor-Specific Gene Expression in Primary Rectal Cancer. Cancers 11, 1142. 10.3390/cancers11081142 31404997 PMC6721540

[B22] Friboulet A. Thomas D. (2005). Systems Biology—an interdisciplinary approach. Biosens. Bioelectron. 20, 2404–2407. 10.1016/j.bios.2004.11.014 15854815

[B23] Friebel E. Kapolou K. Unger S. Núñez N. G. Utz S. Rushing E. J. (2020). Single-Cell Mapping of Human Brain Cancer Reveals Tumor-Specific Instruction of Tissue-Invading Leukocytes. Cell 181, 1626–1642. 10.1016/j.cell.2020.04.055 32470397

[B24] Granja J. M. Corces M. R. Pierce S. E. Bagdatli S. T. Choudhry H. Chang H. Y. (2021). ArchR is a scalable software package for integrative single-cell chromatin accessibility analysis. Nat. Genet. 53, 403–411. 10.1038/s41588-021-00790-6 33633365 PMC8012210

[B25] Gaw G. Gribben M. (2022). Can we detect biomarkers of oral squamous cell carcinoma from saliva or mouth swabs? Evid. Based Dent. 23, 32–33. 10.1038/s41432-022-0248-9 35338327

[B26] Gorris M. A. J. Halilovic A. Rabold K. van Duffelen A. Wickramasinghe I. N. Verweij D. (2018). Eight-Color Multiplex Immunohistochemistry for Simultaneous Detection of Multiple Immune Checkpoint Molecules within the Tumor Microenvironment. J. Immunol. 200, 347–354. 10.4049/jimmunol.1701262 29141863

[B27] Hansen J. Sealfon R. Menon R. Eadon M. T. Lake B. B. Steck B. (2022). A reference tissue atlas for the human kidney. Sci. Adv. 8, eabn4965. 10.1126/sciadv.abn4965 35675394 PMC9176741

[B28] Haug K. Salek R. M. Conesa P. Hastings J. de Matos P. Rijnbeek M. (2013). MetaboLights—an open-access general-purpose repository for metabolomics studies and associated meta-data. Nucleic Acids Res. 41, D781–D786. 10.1093/nar/gks1004 23109552 PMC3531110

[B29] He S. Bhatt R. Brown C. Brown E. A. Buhr D. L. Chantranuvatana K. (2022). High-plex imaging of RNA and proteins at subcellular resolution in fixed tissue by spatial molecular imaging. Nat. Biotechnol. 40, 1794–1806. 10.1038/s41587-022-01483-z 36203011

[B30] Heindl A. Nawaz S. Yuan Y. (2015). Mapping spatial heterogeneity in the tumor microenvironment: a new era for digital pathology. Lab. Invest. 95, 377–384. 10.1038/labinvest.2014.155 25599534

[B31] Heo Y. J. Hwa C. Lee G.-H. Park J.-M. An J.-Y. (2021). Integrative Multi-Omics Approaches in Cancer Research: from Biological Networks to Clinical Subtypes. Mol. Cells 44, 433–443. 10.14348/molcells.2021.0042 34238766 PMC8334347

[B32] Hicks S. A. Strümke I. Thambawita V. Hammou M. Riegler M. A. Halvorsen P. (2022). On evaluation metrics for medical applications of artificial intelligence. Sci. Rep. 12, 5979. 10.1038/s41598-022-09954-8 35395867 PMC8993826

[B33] Hoeben A. Joosten E. A. J. van den Beuken-van Everdingen M. H. J. (2021). Personalized Medicine: recent Progress in Cancer Therapy. Cancers 13, 242. 10.3390/cancers13020242 33440729 PMC7826530

[B34] Hsu S.-C. Chang S. Y. Hwang Y. T. Terng H. J. Tsai C. L. Shen C. H. (2023). mRNA markers associated with malignant pleural effusion. Sci. Rep. 13, 6677. 10.1038/s41598-023-32872-2 37095178 PMC10126123

[B35] James G. Witten D. Hastie T. Tibshirani R. (2017). An introduction to statistical learning with applications in R. Springer.

[B36] Kalluri R. Weinberg R. A. (2009). The basics of epithelial-mesenchymal transition. J. Clin. Invest. 119, 1420–1428. 10.1172/JCI39104 19487818 PMC2689101

[B37] Kanehisa M. Goto S. (2000). KEGG: kyoto Encyclopedia of Genes and Genomes. Nucleic Acids Res. 28, 27–30. 10.1093/nar/28.1.27 10592173 PMC102409

[B38] Karacosta L. G. Anchang B. Ignatiadis N. Kimmey S. C. Benson J. A. Shrager J. B. (2019). Mapping lung cancer epithelial-mesenchymal transition states and trajectories with single-cell resolution. Nat. Commun. 10, 5587. 10.1038/s41467-019-13441-6 31811131 PMC6898514

[B39] Karacosta L. G. (2021). From imaging a single cell to implementing precision medicine: an exciting new era. Emerg. Top. Life Sci. 5, 837–847. 10.1042/ETLS20210219 34889448 PMC8786301

[B40] Kashyap A. Rapsomaniki M. A. Barros V. Fomitcheva-Khartchenko A. Martinelli A. L. Rodriguez A. F. (2022). Quantification of tumor heterogeneity: from data acquisition to metric generation. Trends Biotechnol. 40, 647–676. 10.1016/j.tibtech.2021.11.006 34972597

[B41] Keren L. Bosse M. Marquez D. Angoshtari R. Jain S. Varma S. (2018). A Structured Tumor-Immune Microenvironment in Triple Negative Breast Cancer Revealed by Multiplexed Ion Beam Imaging. Cell 174, 1373–1387. 10.1016/j.cell.2018.08.039 30193111 PMC6132072

[B42] Kim H. Sim M. Park N. Kwon K. Kim J. Kim J. (2022). msPIPE: a pipeline for the analysis and visualization of whole-genome bisulfite sequencing data. BMC Bioinforma. 23, 383. 10.1186/s12859-022-04925-2 PMC9487059 36123620

[B43] Kuett L. Catena R. Özcan A. Plüss A. Cancer Grand Challenges IMAXT Consortium, Schraml, P., et al. (2021). Three-dimensional imaging mass cytometry for highly multiplexed molecular and cellular mapping of tissues and the tumor microenvironment. Nat. Cancer 3, 122–133. 10.1038/s43018-021-00301-w 35121992 PMC7613779

[B44] Langmead B. Salzberg S. L. (2012). Fast gapped-read alignment with Bowtie 2. Nat. Methods 9, 357–359. 10.1038/nmeth.1923 22388286 PMC3322381

[B45] Langreth R. Waldholz M. (1999). New Era of Personalized Medicine: targeting Drugs For Each Unique Genetic Profile. Oncol. 4, 426–427. 10.1634/theoncologist.4-5-426 10551559

[B46] Leinonen R. Sugawara H. Shumway M. International Nucleotide Sequence Database Collaboration (2011). The Sequence Read Archive. Nucleic Acids Res. 39, D19–D21. 10.1093/nar/gkq1019 21062823 PMC3013647

[B47] Li X. Wang C.-Y. (2021). From bulk, single-cell to spatial RNA sequencing. Int. J. Oral Sci. 13, 36–6. 10.1038/s41368-021-00146-0 34782601 PMC8593179

[B48] Lin J.-R. Fallahi-Sichani M. Chen J.-Y. Sorger P. K. (2016). Cyclic Immunofluorescence (CycIF), A Highly Multiplexed Method for Single-cell Imaging. Curr. Protoc. Chem. Biol. 8, 251–264. 10.1002/cpch.14 27925668 PMC5233430

[B49] Liu J. Gao C. Sodicoff J. Kozareva V. Macosko E. Z. Welch J. D. (2020). Jointly defining cell types from multiple single-cell datasets using LIGER. Nat. Protoc. 15, 3632–3662. 10.1038/s41596-020-0391-8 33046898 PMC8132955

[B50] Liu J. Lau S. K. Varma V. A. Moffitt R. A. Caldwell M. Liu T. (2010). Molecular Mapping of Tumor Heterogeneity on Clinical Tissue Specimens with Multiplexed Quantum Dots. ACS Nano 4, 2755–2765. 10.1021/nn100213v 20377268 PMC2923482

[B51] Liu R. Zhang G. Yang Z. (2019). Towards rapid prediction of drug-resistant cancer cell phenotypes: single cell mass spectrometry combined with machine learning. Chem. Commun. 55, 616–619. 10.1039/c8cc08296k PMC6640148 30525135

[B52] Lomakin A. Svedlund J. Strell C. Gataric M. Shmatko A. Rukhovich G. (2022). Spatial genomics maps the structure, nature and evolution of cancer clones. Nature 611, 594–602. 10.1038/s41586-022-05425-2 36352222 PMC9668746

[B53] Luca B. A. Steen C. B. Matusiak M. Azizi A. Varma S. Zhu C. (2021). Atlas of clinically distinct cell states and ecosystems across human solid tumors. Cell 184, 5482–5496.e28. 10.1016/j.cell.2021.09.014 34597583 PMC8526411

[B54] Lukonin I. Zinner M. Liberali P. (2021). Organoids in image-based phenotypic chemical screens. Exp. Mol. Med. 53, 1495–1502. 10.1038/s12276-021-00641-8 34663938 PMC8569209

[B55] Ma Y. Sun Z. Zeng P. Zhang W. Lin Z. (2022). JSNMF enables effective and accurate integrative analysis of single-cell multiomics data. Brief. Bioinform. 23, bbac105. 10.1093/bib/bbac105 35380624

[B56] Maaten L. v. d Hinton G. (2008). Visualizing Data using t-SNE. J. Mach. Learn. Res. 9, 2579–2605.

[B57] MacDonald J. R. Ziman R. Yuen R. K. C. Feuk L. Scherer S. W. (2014). The Database of Genomic Variants: a curated collection of structural variation in the human genome. Nucleic Acids Res. 42, D986–D992. 10.1093/nar/gkt958 24174537 PMC3965079

[B58] Mehrmohamadi M. Sepehri M. H. Nazer N. Norouzi M. R. (2021). A Comparative Overview of Epigenomic Profiling Methods. Front. Cell Dev. Biol. 9, 714687. 10.3389/fcell.2021.714687 34368164 PMC8340004

[B59] Merritt C. R. Ong G. T. Church S. E. Barker K. Danaher P. Geiss G. (2020). Multiplex digital spatial profiling of proteins and RNA in fixed tissue. Nat. Biotechnol. 38, 586–599. 10.1038/s41587-020-0472-9 32393914

[B60] Mintz B. Illmensee K. (1975). Normal genetically mosaic mice produced from malignant teratocarcinoma cells. Proc. Natl. Acad. Sci. 72, 3585–3589. 10.1073/pnas.72.9.3585 1059147 PMC433040

[B61] Mundi P. S. Dela Cruz F. S. Grunn A. Diolaiti D. Mauguen A. Rainey A. R. (2023). A Transcriptome-Based Precision Oncology Platform for Patient-Therapy Alignment in a Diverse Set of Treatment-Resistant Malignancies. Cancer Discov. 13, 1386–1407. 10.1158/2159-8290.CD-22-1020 37061969 PMC10239356

[B62] Neophytou C. M. Panagi M. Stylianopoulos T. Papageorgis P. (2021). The Role of Tumor Microenvironment in Cancer Metastasis: molecular Mechanisms and Therapeutic Opportunities. Cancers 13, 2053. 10.3390/cancers13092053 33922795 PMC8122975

[B63] Park C. C. Bissell M. J. Barcellos-Hoff M. H. (2000). The influence of the microenvironment on the malignant phenotype. Mol. Med. Today 6, 324–329. 10.1016/s1357-4310(00)01756-1 10904250

[B64] Pastushenko I. Brisebarre A. Sifrim A. Fioramonti M. Revenco T. Boumahdi S. (2018). Identification of the tumour transition states occurring during EMT. Nature 556, 463–468. 10.1038/s41586-018-0040-3 29670281

[B65] Patkulkar P. A. Subbalakshmi A. R. Jolly M. K. Sinharay S. (2023). Mapping Spatiotemporal Heterogeneity in Tumor Profiles by Integrating High-Throughput Imaging and Omics Analysis. ACS Omega 8, 6126–6138. 10.1021/acsomega.2c06659 36844580 PMC9948167

[B66] Pfohl U. Pflaume A. Regenbrecht M. Finkler S. Graf Adelmann Q. Reinhard C. (2021). Precision Oncology Beyond Genomics: the Future Is Here—It Is Just Not Evenly Distributed. Cells 10, 928. 10.3390/cells10040928 33920536 PMC8072767

[B67] Proietto M. Crippa M. Damiani C. Pasquale V. Sacco E. Vanoni M. (2023). Tumor heterogeneity: preclinical models, emerging technologies, and future applications. Front. Oncol. 13, 1164535. 10.3389/fonc.2023.1164535 37188201 PMC10175698

[B68] Ptacek J. Locke D. Finck R. Cvijic M. E. Li Z. Tarolli J. G. (2020). Multiplexed ion beam imaging (MIBI) for characterization of the tumor microenvironment across tumor types. Lab. Invest. 100, 1111–1123. 10.1038/s41374-020-0417-4 32203152

[B69] Qin S. Jiang J. Lu Y. Nice E. C. Huang C. Zhang J. (2020). Emerging role of tumor cell plasticity in modifying therapeutic response. Signal Transduct. Target. Ther. 5, 228. 10.1038/s41392-020-00313-5 33028808 PMC7541492

[B70] Randriamanantsoa S. Papargyriou A. Maurer H. C. Peschke K. Schuster M. Zecchin G. (2022). Spatiotemporal dynamics of self-organized branching in pancreas-derived organoids. Nat. Commun. 13, 5219. 10.1038/s41467-022-32806-y 36064947 PMC9445099

[B71] Salgia R. Kulkarni P. (2018). The Genetic/Non-genetic Duality of Drug ‘Resistance’ in Cancer. Trends Cancer 4, 110–118. 10.1016/j.trecan.2018.01.001 29458961 PMC5822736

[B72] Satija R. Farrell J. A. Gennert D. Schier A. F. Regev A. (2015). Spatial reconstruction of single-cell gene expression data. Nat. Biotechnol. 33, 495–502. 10.1038/nbt.3192 25867923 PMC4430369

[B73] Schliekelman M. J. Taguchi A. Zhu J. Dai X. Rodriguez J. Celiktas M. (2015). Molecular Portraits of Epithelial, Mesenchymal, and Hybrid States in Lung Adenocarcinoma and Their Relevance to Survival. Cancer Res. 75, 1789–1800. 10.1158/0008-5472.CAN-14-2535 25744723 PMC4846295

[B74] Shaffer S. M. Dunagin M. C. Torborg S. R. Torre E. A. Emert B. Krepler C. (2017). Rare cell variability and drug-induced reprogramming as a mode of cancer drug resistance. Nature 546, 431–435. 10.1038/nature22794 28607484 PMC5542814

[B75] Sharma S. V. Lee D. Y. Li B. Quinlan M. P. Takahashi F. Maheswaran S. (2010). A Chromatin-Mediated Reversible Drug-Tolerant State in Cancer Cell Subpopulations. Cell 141, 69–80. 10.1016/j.cell.2010.02.027 20371346 PMC2851638

[B76] Siravegna G. Marsoni S. Siena S. Bardelli A. (2017). Integrating liquid biopsies into the management of cancer. Nat. Rev. Clin. Oncol. 14, 531–548. 10.1038/nrclinonc.2017.14 28252003

[B77] Smith C. A. Want E. J. O’Maille G. Abagyan R. Siuzdak G. (2006). XCMS: processing Mass Spectrometry Data for Metabolite Profiling Using Nonlinear Peak Alignment, Matching, and Identification. Anal. Chem. 78, 779–787. 10.1021/ac051437y 16448051

[B78] Sorolla M. A. Sorolla A. Parisi E. Salud A. Porcel J. M. (2021). Diving into the Pleural Fluid: liquid Biopsy for Metastatic Malignant Pleural Effusions. Cancers 13, 2798. 10.3390/cancers13112798 34199799 PMC8200094

[B79] Stack E. C. Wang C. Roman K. A. Hoyt C. C. (2014). Multiplexed immunohistochemistry, imaging, and quantitation: A review, with an assessment of Tyramide signal amplification, multispectral imaging and multiplex analysis. Methods 70, 46–58. 10.1016/j.ymeth.2014.08.016 25242720

[B80] Ståhl P. L. Salmén F. Vickovic S. Lundmark A. Navarro J. F. Magnusson J. (2016). Visualization and analysis of gene expression in tissue sections by spatial transcriptomics. Science 353, 78–82. 10.1126/science.aaf2403 27365449

[B81] Staunstrup N. H. Starnawska A. Nyegaard M. Christiansen L. Nielsen A. L. Børglum A. (2016). Genome-wide DNA methylation profiling with MeDIP-seq using archived dried blood spots. Clin. Epigenetics 8, 81. 10.1186/s13148-016-0242-1 27462375 PMC4960904

[B82] Steen C. B. Luca B. A. Esfahani M. S. Azizi A. Sworder B. J. Nabet B. Y. (2021). The landscape of tumor cell states and ecosystems in diffuse large B cell lymphoma. Cancer Cell 39, 1422–1437.e10. 10.1016/j.ccell.2021.08.011 34597589 PMC9205168

[B83] Street K. Risso D. Fletcher R. B. Das D. Ngai J. Yosef N. (2018). Slingshot: cell lineage and pseudotime inference for single-cell transcriptomics. BMC Genomics 19, 477. 10.1186/s12864-018-4772-0 29914354 PMC6007078

[B84] Svensson V. Teichmann S. A. Stegle O. (2018). SpatialDE: identification of spatially variable genes. Nat. Methods 15, 343–346. 10.1038/nmeth.4636 29553579 PMC6350895

[B85] Tate J. G. Bamford S. Jubb H. C. Sondka Z. Beare D. M. Bindal N. (2019). COSMIC: the Catalogue Of Somatic Mutations In Cancer. Nucleic Acids Res. 47, D941–D947. 10.1093/nar/gky1015 30371878 PMC6323903

[B86] Tian L. Li Y. Edmonson M. N. Zhou X. Newman S. McLeod C. (2020). CICERO: a versatile method for detecting complex and diverse driver fusions using cancer RNA sequencing data. Genome Biol. 21, 126. 10.1186/s13059-020-02043-x 32466770 PMC7325161

[B87] Tóth Z. E. Mezey É. (2007). Simultaneous Visualization of Multiple Antigens with Tyramide Signal Amplification using Antibodies from the same Species. J. Histochem. Cytochem. 55, 545–554. 10.1369/jhc.6A7134.2007 17242468

[B88] Tsao S. C.-H. Wang J. Wang Y. Behren A. Cebon J. Trau M. (2018). Characterising the phenotypic evolution of circulating tumour cells during treatment. Nat. Commun. 9, 1482. 10.1038/s41467-018-03725-8 29662054 PMC5902511

[B89] Vabalas A. Gowen E. Poliakoff E. Casson A. J. (2019). Machine learning algorithm validation with a limited sample size. PLOS ONE 14, e0224365. 10.1371/journal.pone.0224365 31697686 PMC6837442

[B90] Van der Auwera G. A. Carneiro M. O. Hartl C. Poplin R. Del Angel G. Levy-Moonshine A. (2013). From FastQ Data to High-Confidence Variant Calls: the Genome Analysis Toolkit Best Practices Pipeline. Curr. Protoc. Bioinforma. 43, 11. 10.1002/0471250953.bi1110s43 PMC4243306 25431634

[B91] Van Hemelryk A. Erkens-Schulze S. de Ridder C. M. A. Stuurman D. C. Jenster G. W. (2023). Viability Analysis and High-Content Live-Cell Imaging for Drug Testing in Prostate Cancer Xenograft-Derived Organoids. Cells 12, 1377. 10.3390/cells12101377 37408211 PMC10216787

[B92] Vasan N. Baselga J. Hyman D. M. (2019). A view on drug resistance in cancer. Nature 575, 299–309. 10.1038/s41586-019-1730-1 31723286 PMC8008476

[B93] Veenstra T. D. (2021). Omics in Systems Biology: current Progress and Future Outlook. PROTEOMICS 21, 2000235. 10.1002/pmic.202000235 33320441

[B94] Wang M. Carver J. J. Phelan V. V. Sanchez L. M. Garg N. Peng Y. (2016). Sharing and community curation of mass spectrometry data with Global Natural Products Social Molecular Networking. Nat. Biotechnol. 34, 828–837. 10.1038/nbt.3597 27504778 PMC5321674

[B95] Weinstein J. N. Collisson E. A. Mills G. B. Shaw K. R. M. Ozenberger B. A. (2013). The Cancer Genome Atlas Pan-Cancer analysis project. Nat. Genet. 45, 1113–1120. 10.1038/ng.2764 24071849 PMC3919969

[B96] Wellner U. Schubert J. Burk U. C. Schmalhofer O. Zhu F. Sonntag A. (2009). The EMT-activator ZEB1 promotes tumorigenicity by repressing stemness-inhibiting microRNAs. Nat. Cell Biol. 11, 1487–1495. 10.1038/ncb1998 19935649

[B97] Williams C. G. Lee H. J. Asatsuma T. Vento-Tormo R. Haque A. (2022). An introduction to spatial transcriptomics for biomedical research. Genome Med. 14, 68. 10.1186/s13073-022-01075-1 35761361 PMC9238181

[B98] Xia J. Psychogios N. Young N. Wishart D. S. (2009). MetaboAnalyst: a web server for metabolomic data analysis and interpretation. Nucleic Acids Res. 37, W652–W660. 10.1093/nar/gkp356 19429898 PMC2703878

[B99] Xu H. Lien T. Bergholtz H. Fleischer T. Djerroudi L. Vincent-Salomon A. (2021). Multi-Omics Marker Analysis Enables Early Prediction of Breast Tumor Progression. Front. Genet. 12, 670749. 10.3389/fgene.2021.670749 34149812 PMC8209521

[B100] Zhang A. Miao K. Sun H. Deng C.-X. (2022). Tumor heterogeneity reshapes the tumor microenvironment to influence drug resistance. Int. J. Biol. Sci. 18, 3019–3033. 10.7150/ijbs.72534 35541919 PMC9066118

[B101] Zhang J. Rashmi R. Inkman M. Jayachandran K. Ruiz F. Waters M. R. (2021). Integrating imaging and RNA-seq improves outcome prediction in cervical cancer. J. Clin. Invest. 131, e139232. 10.1172/JCI139232 33645544 PMC7919714

[B102] Zhang Y. Liu T. Meyer C. A. Eeckhoute J. Johnson D. S. Bernstein B. E. (2008). Model-based Analysis of ChIP-Seq (MACS). Genome Biol. 9, R137. 10.1186/gb-2008-9-9-r137 18798982 PMC2592715

[B103] Zhang Z. Chng K. R. Lingadahalli S. Chen Z. Liu M. H. Do H. H. (2019). An AR-ERG transcriptional signature defined by long-range chromatin interactomes in prostate cancer cells. Genome Res. 29, 223–235. 10.1101/gr.230243.117 30606742 PMC6360806

[B104] Zhao T. Chiang Z. D. Morriss J. W. LaFave L. M. Murray E. M. Del Priore I. (2022). Spatial genomics enables multi-modal study of clonal heterogeneity in tissues. Nature 601, 85–91. 10.1038/s41586-021-04217-4 34912115 PMC9301586

[B105] Zhao Y. Zhang X. Song Z. Wei D. Wang H. Chen W. (2020). Bibliometric Analysis of ATAC-Seq and Its Use in Cancer Biology via Nucleic Acid Detection. Front. Med. 7, 584728. 10.3389/fmed.2020.584728 PMC7670091 33224964

[B106] Zheng G. X. Y. Terry J. M. Belgrader P. Ryvkin P. Bent Z. W. Wilson R. (2017). Massively parallel digital transcriptional profiling of single cells. Nat. Commun. 8, 14049. 10.1038/ncomms14049 28091601 PMC5241818

[B107] Zheng X. Carstens J. L. Kim J. Scheible M. Kaye J. Sugimoto H. (2015). Epithelial-to-mesenchymal transition is dispensable for metastasis but induces chemoresistance in pancreatic cancer. Nature 527, 525–530. 10.1038/nature16064 26560028 PMC4849281

[B108] Zhu L. Jiang M. Wang H. Sun H. Zhu J. Zhao W. (2021). A narrative review of tumor heterogeneity and challenges to tumor drug therapy. Ann. Transl. Med. 9, 1351. 10.21037/atm-21-1948 34532488 PMC8422119

